# Adaptive responses of cardiorespiratory system and hormonal parameters to individualized high-intensity interval training using anaerobic power reserve in well-trained rowers

**DOI:** 10.3389/fphys.2023.1177108

**Published:** 2023-04-24

**Authors:** Xiaodong Wang, Liqiu Zhao

**Affiliations:** ^1^ School of Physical Education, Shaoguan University, Shaoguan, Guangdong, China; ^2^ Department of Quality Education, Jiangsu Vocational College of Electronics and Information, Huaian, Jiangsu, China

**Keywords:** performance, conditioning, rowing, interval training, individualized intervention

## Abstract

The current study investigated the efficacy of individualizing exercise intensity according to anaerobic power reserve (APR) on hormonal, physiological, and performance adaptations in athletes with different profiles. Sixteen highly-trained male rowers (age = 22 ± 3 years, height = 183 ± 6 cm, weight = 83 ± 7 kg, body fat = 11 ± 2%, experience = 12 ± 5 years) were randomized to a high-intensity interval training consisting of 2 × (6, 6, 8, 8, 10, 10 repetitions from 1st to 6th week, respectively) × 60 s intervals using a rowing ergometer at ∆%30 APR (APR_∆%30_) or the same sets and repetitions at 130% maximal aerobic power (MAP_130%_). In both groups, relief intervals were set at 1:1 with 3 min of rest between sets. On four occasions separated by 24 h recovery, participants attended the laboratory to assess 2000-m rowing ergometer performance, maximal oxygen uptake (V̇O_2_max) and related physiological adaptations, and hormonal parameters. Significant increases were observed in 2000-m performance, V̇O_2_max, ventilation at V̇O_2_max, first and second ventilatory threshold, MAP and maximal sprinting power (MSP), total testosterone, and testosterone to cortisol ratio in response to 6 weeks of APR_∆%30_ and MAP_130%_ protocols. The coefficient of variation (inter-subject variability) in the adaptive response of cardiorespiratory parameters to HIIT performed using the APR_∆%30_ protocol was lower than those of the MAP_130%_ group. However, this is not the case for hormonal changes. Prescribing HIIT based on an athlete’s APR may help to create a more consistent level of the mechanical and physiological stimulus relative to the athlete’s capacity, potentially leading to more similar adaptations across athletes with varying profiles. Mechanisms influencing total testosterone are multifactorial and are not affected by this approach.

## 1 Introduction

The effectiveness of high-intensity interval training (HIIT) in improving physiological, biochemical, and performance adaptations in athletes from a wide range of sports have been unveiled (Laursen and Buchheit, 2019). HIIT programs are prescribed by manipulating variables such as the number of bouts and series, the time and intensity of recovery intervals, and exercise mode (Laursen and Jenkins, 2002). The time and intensity of efforts and relief intervals are the key influencing parameters (Buchheit and Laursen, 2013). The training stimulus should be optimized by conforming these parameters to the athlete’s sports discipline (Du and Tao, 2023). Understanding how to set HIIT variables potentially optimizes adaptive outcomes (Sandford et at., 2021).

The intensity of the exercise period is arguably the first and the most influential characteristic of HIIT sessions (Laursen and Buchheit, 2019). Maximal sprinting power/speed, and critical speed, or maximal lactate steady state, are the high and low ends of the intensity spectrum of HIIT, respectively (Laursen and Buchheit, 2019). Typically, HIIT is prescribed using the power or speed equivalent for maximal oxygen uptake (p/vV̇O_2_max) or maximal sprinting speed/power [MSS/MSP (Julio et al., 2020; Sandford et al., 2021)]. p/vV̇O_2_max is also known as maximal aerobic power/speed [MAP/MAS (Billat and Koralsztein, 1996; Hill and Rowell, 1996)]. Since MAP/MAS is theoretically considered the minimal velocity/power at which V̇O_2_max is elicited (Billat and Koralsztein, 1996), this variable could “represent an ideal reference for training” to improve V̇O_2_max and related cardiorespiratory parameters (Buchheit and Laursen, 2013). MAP/MAS as the reference intensity is suitable for intervals performed around V̇O_2_max, and for the training intensities beyond MAP/MAS, other physiological attributes should be considered. In efforts at supramaximal intensities (i.e., beyond MAP/MAS), optimal responses are related to the degree of anaerobic speed/power reserve (ASR/APR) used rather than the relative intensity concerning MAS (Buchheit and Laursen, 2013; Sandford et al., 2021). APR/ASR is defined as the difference between an individual’s MAP/MAS and MSS/MSP.

In practice, the ability of MSS/MSP in athletes with the same MAP/MAS could be different (Buchheit and Laursen, 2013). Hence, when performing HIIT at supramaximal intensities, if a similar percentage of MAP/MAS is applied for the athletes, different proportions of ASR/APR will be involved, which results in different exercise tolerance, physiological demands, and adaptations across individuals (Blondel et al., 2001; Sandford et al., 2021). Theoretically, when the intensity is above MAP/MAS, it is better to express exercise tolerance as %ASR/APR to diminish inter-individual variance (Blondel et al., 2001; Sandford et al., 2021). To support this, Camus et al. (1988) have shown that expressing exercise intensity with regard to “the difference between the energy required to run at a selected supramaximal velocity minus the maximal aerobic energy expenditure” will reduce variability in exercise tolerance in supramaximal events. Collision et al. (2022) have recently indicated that individualizing running speed as a proportion of ASR in supramaximal intervals reduces inter-subject variability in running performance.

Although evidence indicates that exercise intensity as a proportion of ASR/APR imposes more constant physiological stress and aids in similar physiological demands across individuals, it is unclear if such an approach will result in more homogenized subsequent adaptations across athletes with different ASR/APR. Recently, Du and Tao. (2023) have shown that paddling HIIT prescribed using ASR decreases inter-individual variability in physiological adaptations among athletes with different profiles. A limitation of their study was that they compared the outcomes in response to the maximal (vV̇O_2_max) with a supramaximal intensity. Besides, the significance of hormone production in facilitating the body’s adaptation to HIIT is crucial (Hoff and Triplett, 2016). During HIIT a cascade of hormones is released and the absolute secretion rates of many hormones is augmented (Hoff and Triplett, 2016). Hormonal response is thought to be triggered by the metabolic stress induced by HIIT affecting integrity of muscle, bone, and connective tissue as well as assist in maintaining metabolism within a normal range (Farzad et al., 2011; Konopka and Harber, 2014; Hoff and Triplett, 2016). The magnitude of the hormonal response to HIIT can vary depending on factors such as exercise intensity, duration, and frequency (Sheykhlouvand et al., 2016) and it is unclear if the mentioned uniform adaptations through individualized HIIT using MSP would also be seen in hormonal changes. Therefore, the current study aimed to examine if individualizing the intensity of supramaximal HIIT according to the athlete’s MSP would decrease inter-individual variability in hormonal and physiological adaptive responses compared to the HIIT intervention prescribed using MAP in well-trained rowers. We hypothesized that supramaximal HIIT based on APR would result in more uniform adaptations.

## 2 Materials and methods

### 2.1 Participants

Twenty national-level male rowers voluntarily participated after signing a written informed consent. Participants were members of a national-level club. Following an announcement, interested participants volunteered, and according to a classification framework provided by McKay et al. (2022), eligible rowers were included. The participants’ mean ± SD characteristics were as follows: age = 22 ± 3 years, height = 183 ± 6 cm, weight = 83 ± 7 kg, body fat = 11 ± 2%, experience = 12 ± 5 years. Participants were medication-free and, after the medical screening, were randomized to two different HIIT groups of 10 participants. All procedures were according to the ethical standards of the Helsinki Declaration and were approved by the ethical committee of Shaoguan University, China.

### 2.2 Experimental design

A randomized control design consisted of two experimental groups with four testing sessions pre- and post-training and a 6-week training period. In both pre-and post-training and on four occasions separated by 24 h recovery, participants attended the lab to assess hormonal and physiological parameters and time trial performance. They were asked not to engage in strenuous exercise and to consume the same diet in the 24 h period preceding the testing sessions and refrain from caffein and alcohol (Barzegar et al., 2021). Using a rowing ergometer (Concept2 RowErg^®^, Morristown, Vermont, United States), participants completed a 2000-m time trial. On a particular day, they underwent an incremental exercise using the mentioned rowing ergometer to assess cardiorespiratory system parameters by a gas collection system (METALYZER^®^ 3B, Cortex, Leipzig, Germany). In the third and fourth visits 24 h apart, a maximal sprinting power test and resting blood sampling were done to evaluate MSP and hormonal parameters, respectively. 48 h after finishing the pre-tests, the first session of the 6-week training period was initiated, and 48 h after the last training session, under similar conditions and in the same order, the participants repeated the same testing sessions.

### 2.3 2000-m time trial

Using the same air-braked ergometer (Concept2), participants completed a 2000-m TT per the standard procedure. Before the test, the drag factor of the ergometer was applied, corresponding to the participant’s weight category. Participants completed the test after a 10 min warm-up with a self-selected pace and stretches, and performance time was recorded (Gharaat et al., 2020).

### 2.4 Incremental exercise test

The incremental exercise test followed a previously described protocol (Mikulic, 2009; Faelli et al., 2022). Following a standardized warm-up consisting of 4, 3, and 3 min rowing at 120, 150, and 120 W, respectively, and self-selected stretches, the test began with 3 min rowing at 150 W and then by 25 W increase in workload every 1 minute. The gas collection system continuously measured the cardiorespiratory variables and was calibrated by an experienced technician before each test. The V̇O_2_peak was established as the highest 30 s average of the V̇O_2_ values. V̇O_2_max was verified if at least three of the following criteria were met: a) plateau or a slight drop in V̇O_2_ despite increasing workload; B) respiratory exchange ratio (RER) exceeding 1.1; C) attaining ≥90% age-predicted heart rate; D) visible exhaustion (Fereshtian et al., 2017; Faelli et al., 2022; Sheykhlouvand et al., 2022). Power at V̇O_2_max (MAP) was defined as the minimal power at which V̇O_2_max occurred, provided it could be maintained for 1 minute. Otherwise, power corresponding to the previous stage was recorded as MAP (Sheykhlouvand et al., 2018; Faelli et al., 2022). Oxygen pulse (V̇O_2_/HR), respiratory rate (R*f*), tidal volume (V̇_T_), and ventilation (V̇_E_) at V̇O_2_max were also measured using a gas collection system. The second ventilatory threshold (VT_2_) localized by two independent experts and criterion was the continuous increase in the V̇_E_ equivalent for O_2_ (V̇_E_ V̇O_2_^−1^) and the V̇_E_ equivalent for CO_2_ (V̇_E_ V̇CO_2_^−1^) ratio curves related to the reduction in end-tidal O_2_ tension (P_ET_O_2_). The first ventilatory threshold (VT_1_) was also identified as the point where an elevation in V̇_E_ V̇O_2_^−1^ and P_ET_O_2_ occurred with no simultaneous increase in the V̇_E_ V̇CO_2_^−1^ (Alejo et al., 2022).

### 2.5 Maximal sprinting power and anaerobic power reserve

To assess MSP, participants completed an all-out test using the ergometer. Following a standardized warm-up like the one completed before the incremental exercise test, they were instructed to perform it at the maximal stroke rate and reach their maximum within ten strokes. Power output was tracked stroke-by-stroke, and the mean value of five final strokes were considered MSP (Stevens et al., 2015; Chéilleachair et al., 2017). APR was calculated as follows:

APR=MSP – MAP

### 2.6 Blood sampling and analysis

Following overnight fasting exceeding 8 hours, participants arrived at the laboratory between 6 and 8 a.m. Ten milliliters of blood was collected from the antecubital vein. The blood sample was spun at 3,000 rpm for 15 min at 4 °C and stored at −80 °C until subsequent analysis. Serum cortisol concentrations [CD creative diagnostics, NY, United States; intra-assay CV = 9.32%] and testosterone [Eagle Biosciences, Nashua, NH, United States; intra-assay CV = 6.6%] were analyzed using enzyme-linked immunoassay kits.

### 2.7 Training programs

48 h after the baseline measurements, participants underwent HIIT 3 days per week based on their APR (APR_∆%30_) or MAP (MAP_130%_) for 6 weeks. In the APR_∆%30_ group, participants performed two series of 60 s interval efforts at ∆%30 APR with incremental volume every 2 weeks [6, 6, 8, 8, 10, 10 (repetitions/each set from the 1^st^ to the 6^th^ week)]. The intensity of APR_∆%30_ was calculated as follows:

∆%30APR=MAP+0.3×APR

In the MAP_130%_ group, participants completed two series of 60 s interval efforts at 130% MAP with incremental volume every 2 weeks [6, 6, 8, 8, 10, 10 (repetitions/set from the 1^st^ to the 6^th^ week)]. In both groups, relief intervals were set at 1:1 with 3 min of rest between sets.

### 2.8 Statistical analysis

**FIGURE 1:**
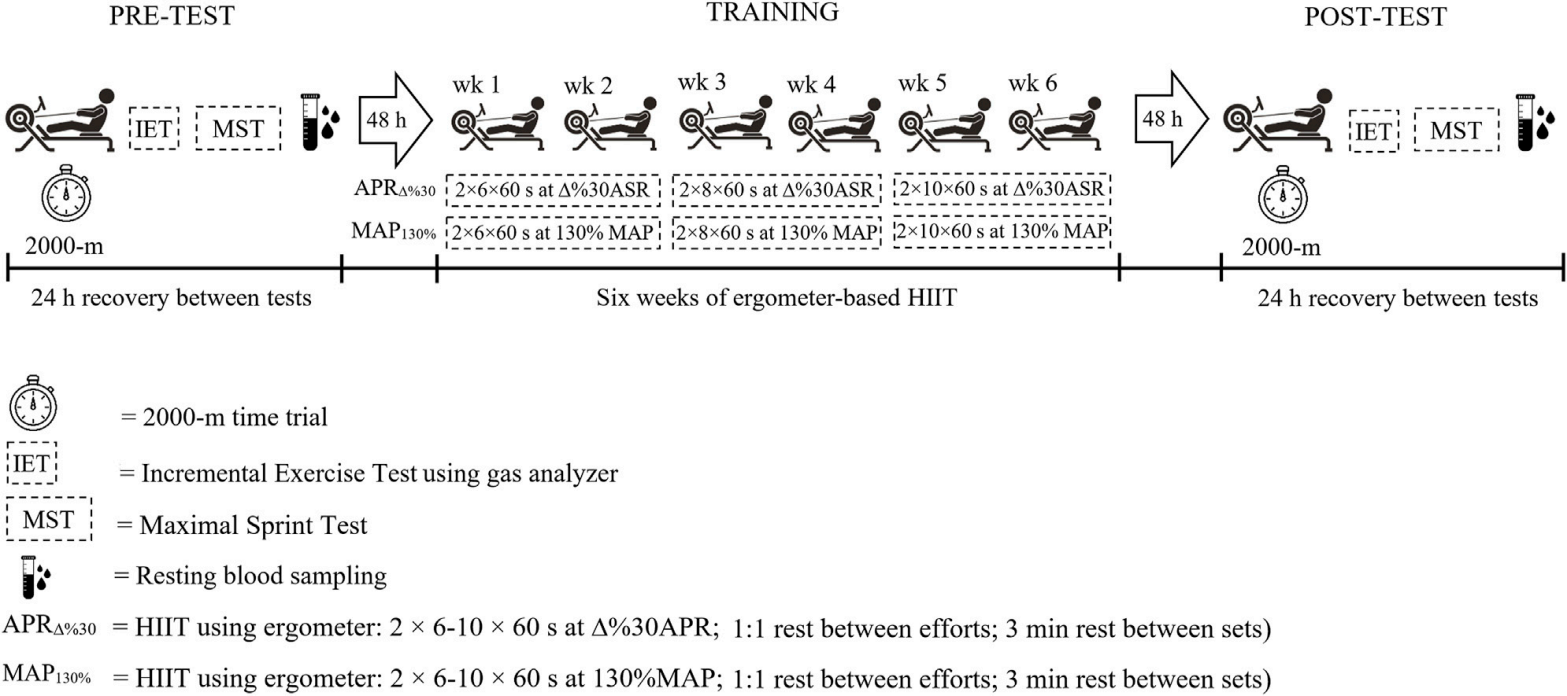
Overview of the experimental protocol.

The sample size was estimated using G*Power software (Faul et al., 2007). Using an effect size of 0.8 and assuming alpha error of 0.05 and β of 0.08, sample size was estimated to be at least six participants in each group. However, the sample size was later increased to 10 participants per group, taking into consideration the possibility of some participants dropping out during data collection. IBM SPSS software version 21 (IBM Corp., Chicago, IL) was used for statistical analysis and reporting data expressed as mean ± SD. Figure 1. The Shapiro-Wilk test and Levene’s test were used to assess the normality of distribution and the homogeneity of variances, respectively. Mauchly’s test analyzed sphericity. A two-factor (group × time) mixed analysis of variance analyzed the between groups difference in the changes, and significance was set at *p* ≤ 0.05. Tukey’s *post hoc* test analyzed the significant interactions or main effects when a significant F-ratio was observed. The effect size was calculated using Cohen’s d (*d*) with small (d = 0.2), medium (d = 0.5), and large (d = 0.8) benchmarks suggested by Cohen. (1988).

## 3 Results

### 3.1 Gas exchange variables

**FIGURE 2:**
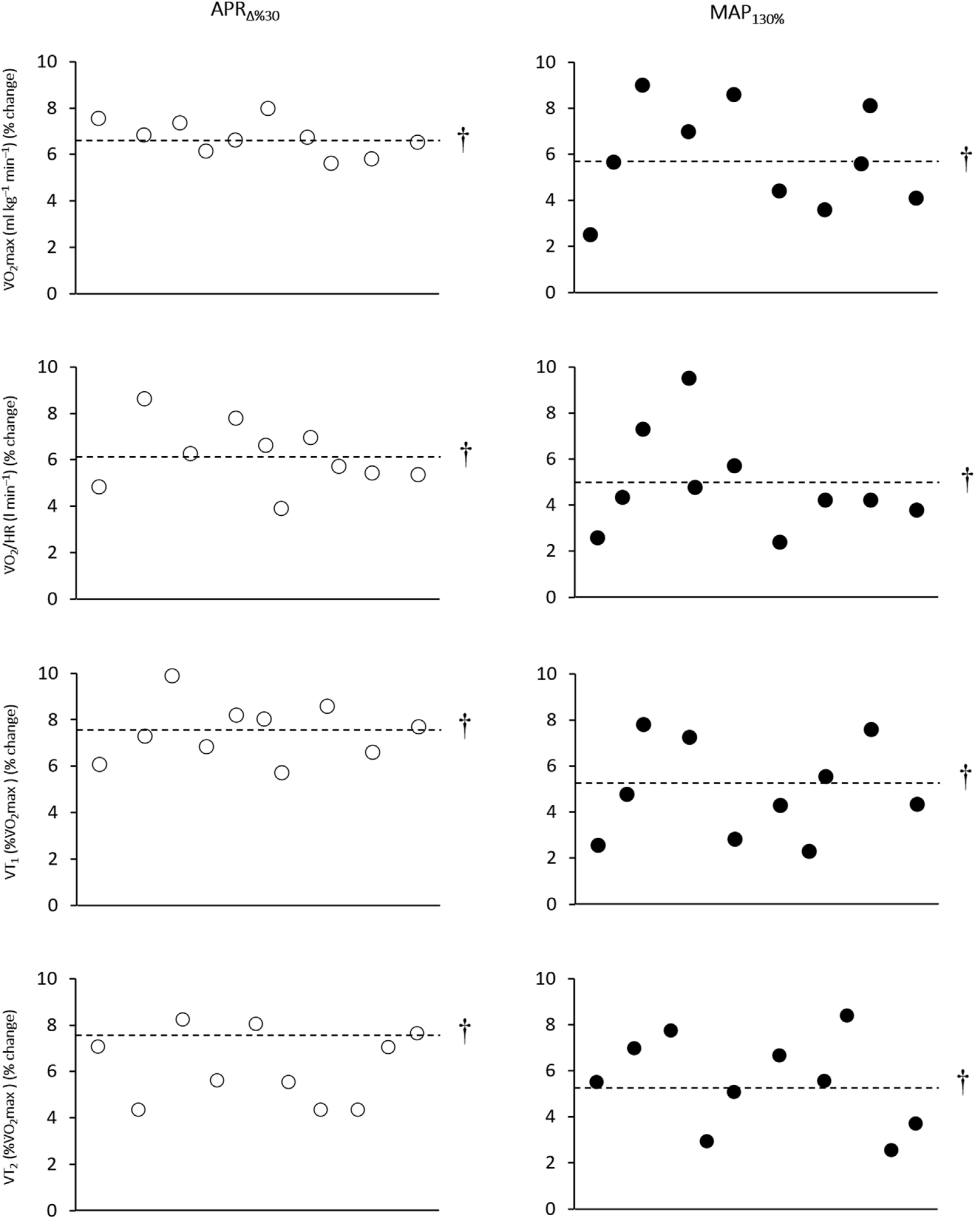
Effects of HIIT based on anaerobic power reserve (APR_∆%30_) and maximal aerobic power (MAP_130%_) on V̇O_2_max, V̇O_2_/HR, VT_1_, and VT_2_. Circles indicate individual percent change from baseline and the dashed line represents the mean change of participants over time. † Denotes Significantly different *versus* pre-training (*p* ≤ 0.05).

Physiological variables showed no between-group difference at the baseline. Relative (ml kg^–1^ min^–1^) and absolute (l min^–1^) V̇O_2_max significantly improved over time in response to 6 weeks of APR_∆%30_ (Pre: 55.4 ± 2.5 vs. 59.1 ± 2.5 ml kg^–1^ min^–1^, %change = 6.6, *p* = 0.005, *d* = 1.4; Pre: 4.452 ± 0.276 vs. 4.763 ± 0.314 l min^–1^, %change = 6.9, *p* = 0.001, *d* = 1.1) and MAP_130%_ (Pre: 54.0 ± 2.6 vs. 57.1 ± 2.9 ml kg^–1^ min^–1^, %change = 5.7, *p* = 0.003, *d* = 1.1; Pre: 4.337 ± 0.217 vs. 4.558 ± 0.203 l min^–1^, %change = 5.09, *p* = 0.002, *d* = 1.0) groups. No between-group difference was observed over time (Figure 2).

Six weeks of APR_∆%30_ significantly enhanced V̇O_2_/HR (Pre: 24.6 ± 1.8 vs. 26.1 ± 1.9 ml b^–1^ min^–1^, %change = 6.1, *p* = 0.001, *d* = 0.8), V̇_E_ at V̇O_2_max (Pre: 186.8 ± 28.2 vs. 212.8 ± 29.3 l min^–1^, %change = 13.9, *p* = 0.003, *d* = 0.9), R_*f*_ at V̇O_2_max (Pre: 70.3 ± 15.7 vs. 80.3 ± 17.2 l min^–1^, %change = 14.2, *p* = 0.002, *d* = 0.7), VT_1_ (Pre: 71.5 ± 8.0 vs. 76.9 ± 7.4 b min^–1^, %change = 7.5, *p* = 0.002, *d* = 0.6), and VT_2_ (Pre: 88.1 ± 5.1 vs. 92.8 ± 3.6 %V̇O_2_max, %change = 5.3, *p* = 0.002, *d* = 1.1) (Figure 2).

Also, MAP_130%_ significantly improved V̇O_2_/HR (Pre: 23.8 ± 1.5 vs. 25.0 ± 1.2 ml b^–1^ min^–1^, %change = 5.0, *p* = 0.003, *d* = 0.7), V̇_E_ at V̇O_2_max (Pre: 185.8 ± 19.1 vs. 212.3 ± 30.8 l min^–1^, %change = 14.2, *p* = 0.003, *d* = 1.0), R_*f*_ at V̇O_2_max (Pre: 67.2 ± 11.2 vs. 77.8 ± 16.1 l min^–1^, %change = 15.7, *p* = 0.002, *d* = 0.8), VT_1_ (Pre: 69.9 ± 4.3 vs. 73.5 ± 3.3 b min^–1^, %change = 5.2, *p* = 0.001, *d* = 0.9), and VT_2_ (Pre: 84.4 ± 5.2 vs. 89.4 ± 6.3 %V̇O_2_max, %change = 5.9, *p* = 0.005, *d* = 0.8) over time (Figure 2).

Inter-subject variability [coefficient of variation (CV)] for the changes in the relative and absolute V̇O_2_max, V̇O_2_/HR, V̇_E_ at V̇O_2_max, R_*f*_ at V̇O_2_max, and VT_1_ in response to APR_∆%30_ was lower than MAP_130%_ group (Table 2).

### 3.2 MSP, MAP, and APR

**FIGURE 3:**
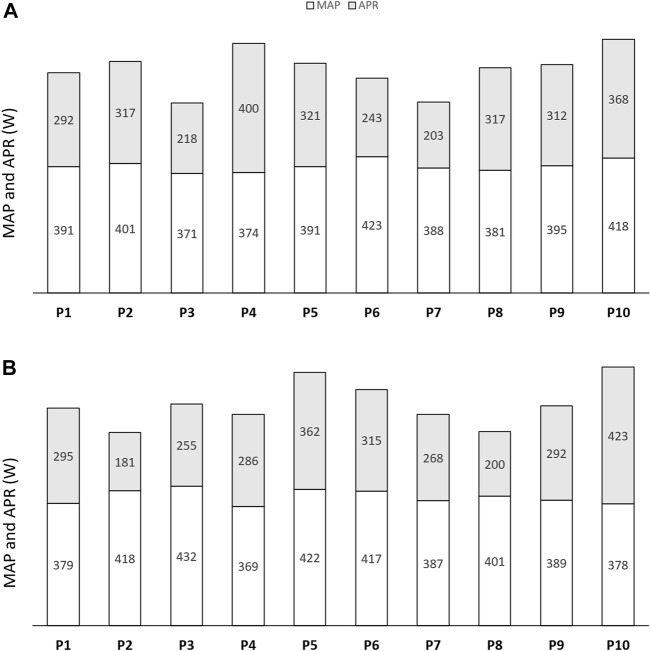
Individual values of maximal aerobic power (MAP) and anaerobic power reserve (APR) in **(A)** APR_∆%30_ group and **(B)** MAP_130%_ group at the baseline.

**FIGURE 4:**
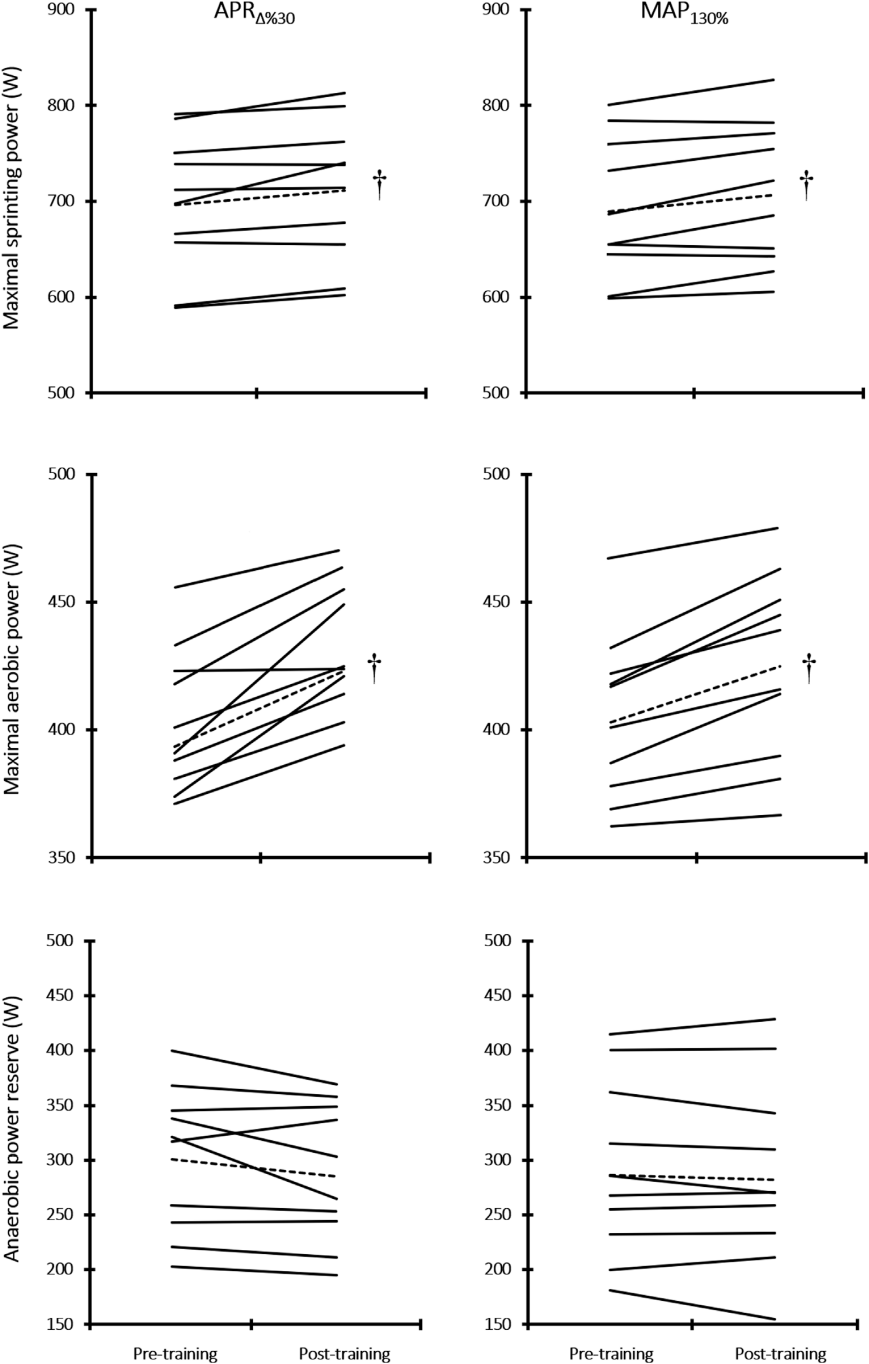
Individual changes in maximal aerobic power (MAP), maximal sprinting power (MSP), and anaerobic power reserve (APR) after the training period. † Denotes Significantly different *versus* pre-training (*p* ≤ 0.05).

Figure 3 indicates individual locomotor abilities (MAP and APR) at the baseline in both APR_∆%30_ and MAP_130%_ groups. Table 1 presents the mean changes from pre-to post-training in both APR_∆%30_ and MAP_130%_ groups; Figure 4 indicates individual changes over time. At the baseline, no difference was observed for the mentioned variables between groups. MSP showed an increasing adaptive response to APR_∆%30_ (*p* = 0.02, *d* = 0.4) and MAP_130%_ (*p* = 0.01, *d* = 0.3) training interventions over time. Also, 6 weeks of HIIT enhanced MAP in both APR_∆%30_ (*p* = 0.002, *d* = 1.5) and MAP_130%_ (*p* = 0.002, *d* = 0.9) groups. HIIT protocols resulted in no significant changes in APR. The CV value for the magnitude of the changes in MAP in response to APR_∆%30_ was lower than the MAP_130%_ group (Table 2).

**TABLE 1:** Maximal sprinting power (MSP) and anaerobic power reserve (APR) before and after HIIT.

	Group					
	APR_∆%30_			MAP_130%_		
	Pre-test	Post-test	%∆	Pre-test	Post-test	%∆
MSP (W)	696.5 ± 37.0	711.6 ± 38.4	† 2.2	689.2 ± 46.1	706.9 ± 48.1	† 2.5
MAP (W)	393.3 ± 19.3	423.1 ± 20.1	† 7.5	399.2 ± 25.2	424.9 ± 29.4	† 6.4
APR (W)	299.1 ± 71.9	284.9 ± 67.1	−5.0	286.2 ± 80.0	278.1 ± 84.9	−2.9

Values are means ± SD

MSP, maximal sprinting power; MAP, maximal aerobic power; APR, anaerobic power reserve.

† *Significantly different from baseline value (p < 0.05)*.

**TABLE 2:** The inter-individual variation (CV) of percent changes in hormonal and physiological variables as well as 2000-m rowing ergometer performance.

	Group			
	APR_∆%30_	MAP_130%_		
	Changes over time	CV	Changes over time	CV
V̇O_2_max (%)	6.9 ± 0.82	0.12	5.7 ± 2.55	0.44
V̇O_2_/HR (%)	6.1 ± 1.57	0.25	5.0 ± 2.40	0.48
V̇_E_ at V̇O_2_max (%)	13.9 ± 4.73	0.34	14.2 ± 8.91	0.62
R*f* at V̇O_2_max (%)	14.2 ± 3.48	0.24	15.7 ± 8.19	0.52
VT_1_ (%)	7.5 ± 1.46	0.19	5.1 ± 2.10	0.41
VT_2_ (%)	5.3 ± 1.62	0.30	5.9 ± 2.41	0.40
MSP (%)	2.2 ± 1.98	0.90	2.5 ± 2.21	0.88
MAP (%)	7.5 ± 4.49	0.59	6.4 ± 4.99	0.77
2000-m TT (%)	–1.5 ± 0.50	0.33	–1.4 ± 0.76	0.54
Total testosterone (%)	10.3 ± 6.01	0.58	12.9 ± 6.74	0.52
T/C ratio (%)	13.4 ± 7.12	0.53	15.4 ± 6.93	0.45

Values are means ± SD

*V̇O*_*2*_ *max, maximum oxygen consumption; HR, heart rate; V̇O*_*2*_ */HR*, *O*_*2*_ *pulse; V̇*_*E*_ *, ventilation, R*_*f*_ *; respiratory rate; VT*_*1*_ *, first ventilatory threshold; VT*_*2*_ *, second ventilatory threshold; MSP, maximal sprinting power; MAP, maximal aerobic power; 2000-m TT, 2000-m rowing ergometer time trial; T/C, testosterone to cortisol ratio; CV, coefficient of variation*.

### 3.3 2000-m ergometer time trial

**FIGURE 5:**
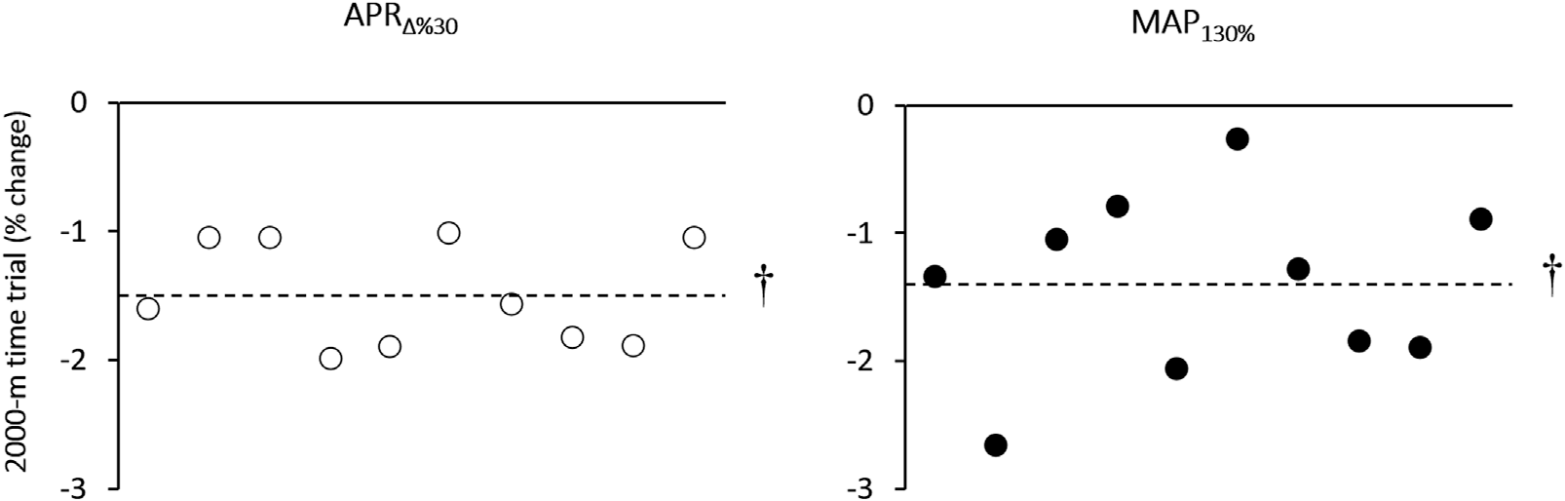
Individual changes in 2000-m rowing ergometer time trial after the training period. † Denotes Significantly different *versus* pre-training (*p* ≤ 0.05).

There was no between-group difference for the 2000-m time trial at the baseline. Figure 5 presents individual changes in 2000-m TT over time. Compared to baseline values, a significant improvement was observed in 2000-m TT in both APR_∆%30_ (Pre: 380.6 ± 2.8 vs. 374.9 ± 3.8 s, change = −5.75 s (−1.5%), *p* = 0.006, *d* = 1.7) and MAP_130%_ (Pre: 377.6 ± 6.1 vs. 372.2 ± 6.0 s, change = −5.40 (−1.4), *p* = 0.001, *d* = 0.9) groups. The magnitude of changes was no difference between groups (*p* > 0.05). CV values for percent changes of the 2000-m ergometer rowing performance in response to APR_∆%30_ were lower than the MAP_130%_ group (Table 2).

### 3.4 Hormonal changes

**FIGURE 6:**
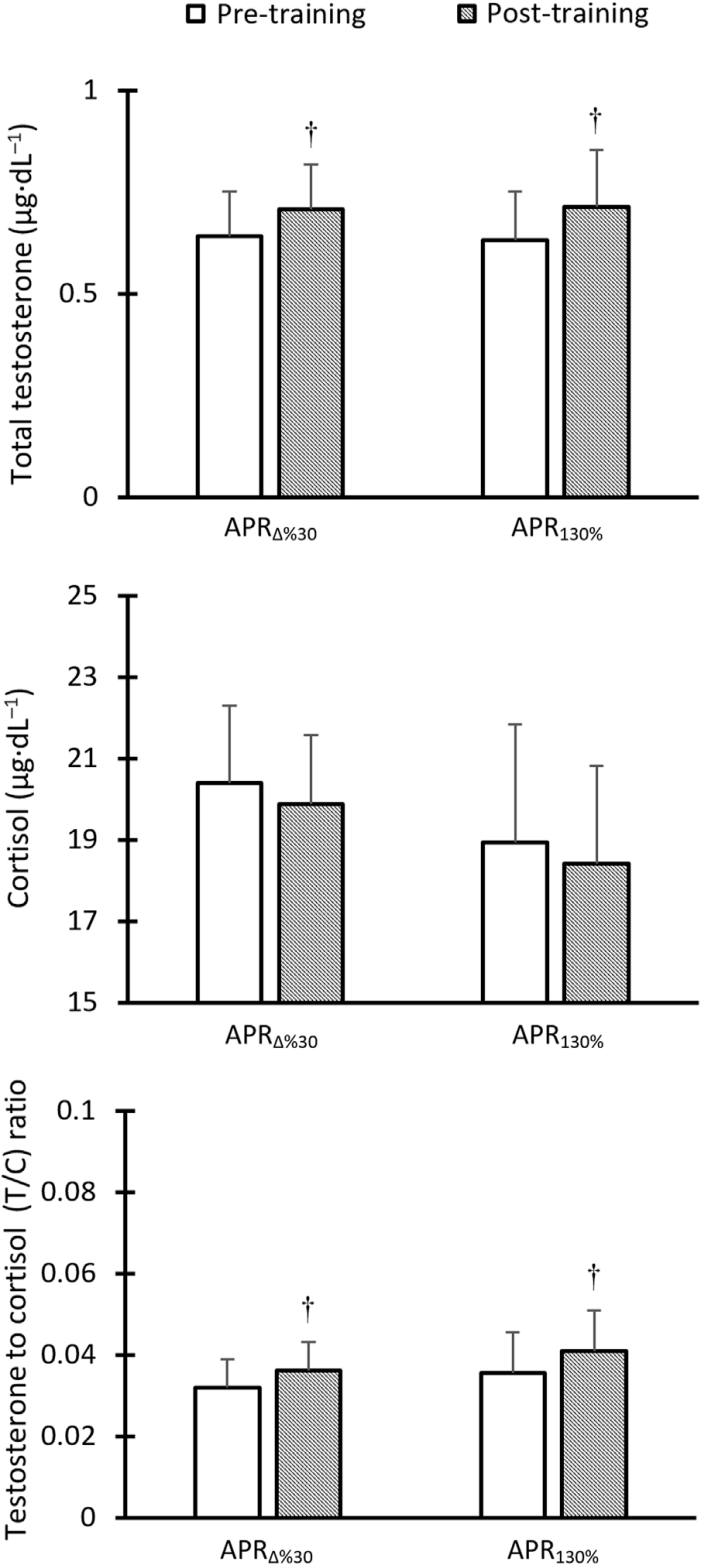
Hormonal changes in response to HIIT based on anaerobic power reserve (APR_∆%30_) and maximal aerobic power (MAP_130%_) over time. † Denotes Significantly different *versus* pre-training (p ≤ 0.05).

Figure 6 presents resting hormonal changes in response to 6 weeks of HIIT. There was no between-group difference at the baseline for total testosterone (TT), cortisol (C), and testosterone to cortisol (T/C) ratio. TT showed a significant increase over time in response to both APR_∆%30_ (Pre: 0.642 ± 0.11 vs. 0.708 ± 0.11 μg dL^–1^, %change = 10.3, *p* = 0.002, *d* = 0.5) and MAP_130%_ (Pre: 0.633 ± 0.13 vs. 0.715 ± 0.14 μg dL^–1^, %change = 12.9, *p* = 0.005, *d* = 0.6) groups. Cortisol levels tended to decrease in both groups by ∼ 2.5%; this was not statistically significant (*p* > 0.05). Also, T/C ratio significantly increased in response to APR_∆%30_ (Pre: 0.0320 ± 0.007 vs. 0.0363 ± 0.007, %change = 13.4, *p* = 0.002, *d* = 0.6) and MAP_130%_ (Pre: 0.0356 ± 0.012 vs. 0.0411 ± 0.013, %change = 15.4, *p* = 0.005, *d* = 0.4). No between-group difference was observed for the magnitude of the changes over time (Figure 6). CV values for percent changes of the TT and T/C ratio in response to APR_∆%30_ and MAP_130%_ interventions were almost the same (Table 2).

## 4 Discussion

This study examined if prescribing supramaximal HIIT based on anaerobic power reserve diminishes inter-individual variability (CV) in the magnitude of adaptations to a 6-week training period among athletes varying in locomotor profiles (APR). The primary findings of this study were that such an approach results in more uniform adaptive responses in physiological parameters and time trial performance in highly-trained rowers. However, this is not the case for hormonal changes. Also, APR_∆%30_ and MAP_130%_ HIIT interventions significantly improved in the mentioned variables.

Only one study has examined the homogeneity of adaptive response to HIIT intervention prescribed using ASR. Our study is the first to test such an approach regarding hormonal changes in highly-trained rowers. Our results support Du and Tao. (2023), who indicated that similar physiological adaptations could be facilitated when HIIT is prescribed using the ASR. Many studies have shown a considerable individual variation in response to standardized programs consisting of exercise interventions prescribed using relative intensity and duration (Mann et al., 2014). have argued such variations in homeostatic stress may affect the magnitude of stimulus experienced by athletes with different profiles and result in different adaptive responses over a training period. The coefficient of variations presented in Table 2 indicates lower values in physiological and performance adaptations in APR_∆%30_ groups compared to MAP_130%_, suggesting HIIT performed based on the athlete’s APR could be considered a successful method to impose equivalent homeostatic stress between individuals and to incur more uniform adaptive response. Such an individualization prevents a mismatch between the athlete’s profile and prescription, and as a result, mechanical and associated physiological stimulus relative to the athlete’s ceilings is normalized (Sandford et al., 2021; Collison et al., 2022; Du and Tao., 2023). In support of this notion, Collison et al., 2022 have shown that when comparing supramaximal interval running trials performed at 120% MAS, 20% ASR, and 30–15 intermittent fitness test, variability of exercise tolerance is decreased. Hence, involving ASR in HIIT supramaximal programs imposes similar physiological stress across individuals and ensures more identical adaptive responses (Collison et al., 2022).

Other important findings of this study were that 6 weeks of supramaximal HIIT performed by APR_∆%30_ and MAP_130%_ protocols significantly improved physiological and performance adaptations in our participants. Adaptive responses to both protocols were almost the same, with a trivial and statistically insignificant difference. Improvements in rowing ergometer performance could be attributed to the enhanced V̇O_2_max, and ventilatory threshold as an indicator of exercise tolerance (Du and Tao, 2023). Increases in V̇O_2_max could also occur through improvements in cardiac function [stroke volume and cardiac output (central component)], and O_2_ utilized by active muscles [peripheral component] as classically proposed (Laursen and Jenkins, 2002; Driller et al., 2009). Elevated V̇O_2_/HR [indirect predictor of stroke volume (Whipp et al., 1996; Laffite et al., 2003)] can be supportive of enhanced central adaptations in response to APR_∆%30_ and MAP_130%_ protocols (Sheykhlouvand et al., 2022; Du and Tao, 2023). Finally, the enhanced T/C ratio in both groups indicate anabolic response to both HIIT interventions.

Hormonal changes over the training period showed almost the same CV value in both training groups with anabolic adaptations to APR_∆%30_ and MAP_130%_ HIIT interventions. We can only speculate on potential mediators responsible for such a response in TT levels. Mechanisms influencing TT are multifactorial, and aside from exercise-induced changes, nutritional, physiological, psychological, environmental, and lifestyle variables such as sleep, food, alcohol, emotions, dominance, and mental fatigue may also affect TT levels (Mathur and D'Cruz, 2011; Ambroży et al., 2021; Zurek et al., 2022). To support this, Mann and others (2014) reported that psychological stress, sleep, as well as composition and timing of diet might also potentially contribute to different adaptive responses. The overall interpretation of the studies mentioned above supports the belief that HIIT tailored to the locomotor profile may result in more similar physiological demands among individuals and lead to more uniform adaptations across diverse athlete profiles (Sandford et al., 2021; Du and Tao, 2023). In fact, exercise tolerance and perceptive and physiological responses at supramaximal intensities is better related to the APR than to MAP (Blondel et al., 2001; Sandford et al., 2021). Athletes with a similar MAP can present with clearly different MSP and APR (Figure 3). If two athletes with similar MAP but varying MSP exercise at the same % of MAP, the exercise will engage different proportions of their APR, leading to varying physiological demands, exercise tolerance, and ultimately different levels of physiological adaptation (Buchheit and Laursen, 2013). Therefore, for individualizing training intensity during supramaximal HIIT, it is essential to consider the measurement of MSP and APR in addition to MAP (Blondel et al., 2001; Sandford et al., 2021). Such an approach facilitates the involvement of the equal proportions of physiological ceiling across individuals with different profiles, reduces supramaximal interval exercise performance variability, ensures similar physiological demand across individuals, and potentially facilitates similar degrees of physiological adaptation (Collision et al., 2022).

A limitation of this study was that although the training time accumulated in HIIT sessions was the same in both groups, the intensity of HIIT bouts was not precisely matched. Another limitation of this study was the lack of strict monitoring of the athletes’ diet, sleep quality, and motivation during the testing sessions. The instructions provided to the participants were aimed at ensuring consistency across the study. However, it was not possible to ideally monitor all the factors. The participants were asked to abstain from caffeine and alcohol, avoid rigorous physical activity the day before the test, and fast for at least 8 hours before morning blood sampling. Nonetheless, complete adherence to all these instructions could not be guaranteed. A strength of this study was using a randomized controlled trial. We also recruited highly trained athletes and we individualized the training using a new method. We also tried to have a comprehensive assessment by evaluating physiological and performance parameters and assessing hormonal factors.

## 5 Conclusion

In conclusion, our results suggest that 6 weeks of HIIT prescribed according to the athlete’s anaerobic power reserve ensures similar physiological demands among individuals with different locomotor abilities and causes a more uniform adaptive response. This is not the case for adaptive response in hormonal parameters. Athletes’ APR may help rowers and their coaches choose more individualized training and such an accurately prescribed interventions helps achieve desired physiological adaptations. Also, HIIT performed using APR_∆%30_ and MAP_130%_ protocols improved aerobic power, ventilatory threshold, 2000-m ergometer rowing performance, and hormonal adaptations in highly-trained rowers under the conditions of this study.

## Data Availability

The raw data supporting the conclusion of this article will be made available by the authors, without undue reservation.

## References

[B1] Alejo L. B. Montalvo-Pérez A. Valenzuela P. L. Revuelta C. Ozcoidi L. M. de la Calle V. (2022). Comparative analysis of endurance, strength and body composition indicators in professional, under-23 and junior cyclists. Front. Physiol. 13, 945552. 10.3389/fphys.2022.945552 35991188 PMC9388719

[B2] Ambroży T. Rydzik Ł. Obmiński Z. Błach W. Błach B. (2021). The effect of high-intensity interval training periods on morning serum testosterone and cortisol levels and physical fitness in men aged 35–40 years. J. Clin. Med. 10 (10), 2143. 10.3390/jcm101021433390/jcm10102143 34063524 PMC8156527

[B3] Barzegar H. Arazi H. Mohsebbi H. Sheykhlouvand M. Forbes S. C. (2021). Caffeine co-ingested with carbohydrate on performance recovery in national level paddlers: A randomized, double-blind, crossover, placebo-controlled trial. J. Sport. Med. Phys. Fit. 62, 337–342. 10.23736/S0022-4707.21.12125-5 34498818

[B4] Billat L. V. Koralsztein J. P. (1996). Significance of the velocity at VO_2_max and time to exhaustion at this velocity. Sports. Med. 22 (2), 90–108. 10.2165/00007256-199622020-00004 8857705

[B5] Blondel N. Berthoin S. Billat V. Lensel G. (2001). Relationship between run times to exhaustion at 90, 100, 120, and 140% of vVO2max and velocity expressed relatively to critical velocity and maximal velocity. Int. J. Sports. Med. 22 (1), 27–33. 10.1055/s-2001-11357 11258638

[B6] Buchheit M. Laursen P. B. (2013). High-intensity interval training, solutions to the programming puzzle: Part I: Cardiopulmonary emphasis. Sports. Med. 43, 313–338. 10.1007/s40279-013-0029-x 23539308

[B7] Camus G. Juchmes J. Thys H. Fossion A. (1988). Relation between endurance time and maximal oxygen consumption during supramaximal running. J. Physiol. Paris. 83, 26–31. 3183977

[B8] Chéilleachair N. J. N. Harrison A. J. Warrington A. G. D. (2017). HIIT enhances endurance performance and aerobic characteristics more than high-volume training in trained rowers. J. Sports. Sci. 35 (11), 1052–1058. 10.1080/02640414.2016.1209539 27438378

[B9] Cohen J. (1988). Statistical power analysis for the behavioral sciences. New York, NY: Routledge Academic.

[B10] Collison J. Debenedictis T. Fuller J. T. Gerschwitz R. Ling T. Gotch L. (2022). Supramaximal interval running prescription in Australian rules football players: A comparison between maximal aerobic speed, anaerobic speed reserve, and the 30-15 intermittent fitness test. J. Strength. Cond. Res. 36 (12), 3409–3414. 10.1519/JSC.0000000000004103 34387223

[B35] Driller M. W. Fell J. W. Gregory J. R. Shing C.M. Williams A. D. (2009). The effects of high-intensity interval training in well-trained rowers. Int. J. Sports. Physiol. Perform. 4, 110–121. 10.1123/ijspp.4.1.110 19417232

[B11] Du G. Tao T. (2023). Effects of a paddling-based high-intensity interval training prescribed using anaerobic speed reserve on sprint kayak performance. Front. Physiol. 13, 1077172. 10.3389/fphys.2022.1077172 36685190 PMC9848400

[B12] Faelli E. Panascì M. Ferrando V. Codella R. Bisio A. Ruggeri P. (2022). High-intensity interval training for rowing: Acute responses in national-level adolescent males. Int. J. Environ. Res. Public. Health. 19 (13), 8132. 10.3390/ijerph19138132 35805789 PMC9265424

[B13] Farzad B. Gharakhanlou R. Agha-Alinejad H. Curby D. G. Bayati M. Bahraminejad M. (2011). Physiological and performance changes from the addition of a sprint interval program to wrestling training. J. Strength. Cond. Res. 25, 2392–2399. 10.1519/JSC.0b013e3181fb4a33 21849912

[B14] Faul F. Erdfelder E. Lang A. G. Buchner A. (2007). G*Power 3: A flexible statistical power analysis program for the social, behavioral, and biomedical sciences. Behav. Res. Methods. 39, 175–191. 10.3758/bf03193146 17695343

[B15] Fereshtian S. Sheykhlouvand M. Forbes S. Agha-Alinejad H. Gharaat M. (2017). Physiological and performance responses to high-intensity interval training in female inline speed skaters. Apunts. Med. L’esport. 52, 131–138. 10.1016/j.apunts.2017.06.003

[B16] Gharaat M. A. Sheykhlouvand M. Eidi L. A. (2020). Performance and recovery: Effects of caffeine on a 2000-m rowing ergometer. Sport. Sci. Health. 16, 531–542. 10.1007/s11332-020-00643-5

[B17] Haff G. G. Triplett N. T. (2016). Essentials of strength training and conditioning. 4th Edn. Champaign, IL: Human Kinetics, 123–124.

[B18] Hill D. W. Rowell A. L. (1996). Running velocity at VO_2_max. Med. Sci. Sports. Exerc. 28 (1), 114–119. 10.1097/00005768-199601000-00022 8775363

[B19] Julio U. F. Valéria L. G. Panissa V. L. G. Paludo A. C. Alves E. D. Campos F. A. D. (2020). Use of the anaerobic speed reserve to normalize the prescription of high-intensity interval exercise intensity. Eur. J. Sport. Sci. 20 (2), 166–173. 10.1080/17461391.2019.1624833 31132025

[B20] Konopka A. R. Harber M. P. (2014). Skeletal muscle hypertrophy after aerobic exercise training. Exerc. Sport. Sci. Rev. 42, 53–61. 10.1249/JES.0000000000000007 24508740 PMC4523889

[B21] Laffite L. P. Mille-Hamard L. Koralsztein J. P. Billat V. L. (2003). The effects of interval training on oxygen pulse and performance in supra-threshold runs. Arch. Physiol. Biochem. 111, 202–210. 10.1076/apab.111.3.202.23455 14972740

[B22] Laursen P. B. Buchheit M. (2019). Science and application of high-intensity interval training. 1st Edn. Champaign: Human Kinetics, 225–457.

[B23] Laursen P. B. Jenkins D. G. (2002). The scientific basis for high-intensity interval training: Optimising training programmes and maximising performance in highly trained endurance athletes. Sports Med. 32 (1), 53–73. 10.2165/00007256-200232010-00003 11772161

[B24] Mathur P. P. D'Cruz S. C. (2011). The effect of environmental contaminants on testicular function. Asian J. Androl. 13 (4), 585–591. 10.1038/aja.2011.40 21706039 PMC3739630

[B34] Mann T. N. Lamberts R. P. Lamberts M. I. (2014). High responders and low responders: factors associated with individual variation in response to standardized training. Sports. Med. 44 (8), 1113–1124. 10.1007/s40279-014-0197-3 24807838

[B25] McKay A. K. A. Stellingwerff T. Smith E. S. Martin D. T. Mujika I. Goosey-Tolfrey V. L. (2022). Defining training and performance caliber: A participant classification framework. Int. J. Sports. Physiol. Perform. 17 (2), 317–331. 10.1123/ijspp.2021-0451 34965513

[B26] Mikulic P. (2009). Anthropometric and metabolic determinants of 6,000-m rowing ergometer performance in internationally competitive rowers. J. Strength. Cond. Res. 23 (6), 1851–1857. 10.1519/JSC.0b013e3181b3dc7e 19675473

[B27] Sandford G. N. Laursen P. B. Buchheit M. (2021). Anaerobic speed/power reserve and sport performance: Scientific basis, current applications and future directions. Sports. Med. 51 (10), 2017–2028. 10.1007/s40279-021-01523-9 34398445

[B28] Sheykhlouvand M. Arazi H. Astorino T. A. Suzuki K. (2022). Effects of a new form of resistance-type high-intensity interval training on cardiac structure, hemodynamics, and physiological and performance adaptations in well-trained kayak sprint athletes. Front. Physiol. 13, 850768. 10.3389/fphys.2022.850768 35360225 PMC8960736

[B29] Sheykhlouvand M. Khalili E. Agha-Alinejad H. Gharaat M. A. (2016). Hormonal and physiological adaptations to high-intensity interval training in professional male canoe polo athletes. J. Strength. Cond. Res. 30, 859–866. 10.1519/JSC.0000000000001161 26349044

[B30] Sheykhlouvand M. Khalili E. Gharaat M. Arazi H. Khalafi M. Tarverdizadeh B. (2018). Practical model of low-volume paddling-based sprint interval training improves aerobic and anaerobic performances in professional female canoe polo athletes. J. Strength. Cond. Res. 32, 2375–2382. 10.1519/JSC.0000000000002152 29239986

[B31] Stevens A. W. J. Olver T. T. Lemon P. W. R. (2015). Incorporating sprint training with endurance training improves anaerobic capacity and 2,000-m Erg performance in trained oarsmen. J. Strength. Cond. Res. 29 (1), 22–28. 10.1519/JSC.0000000000000593 24978833

[B32] Whipp B. J. Higgenbotham M. B. Cobb F. C. (1996). Estimating exercise stroke volume from asymptotic oxygen pulse in humans. J. Appl. Physiol. 81, 2674–2679. 10.1152/jappl.1996.81.6.2674 9018521

[B33] Zurek G. Żurek A. Nowak-Kornicka J. Żelaźniewicz A. Orzechowski S. (2022). Effects of dominance and sprint interval exercise on testosterone and cortisol levels in strength-endurance-and non-training men. Biol. (Basel) 11 (7), 961. 10.3390/biology11070961 PMC9312330 36101342

